# Synergistic Effect of Nano-Sliver with Sucrose on Extending Vase Life of the Carnation cv. Edun

**DOI:** 10.3389/fpls.2017.01601

**Published:** 2017-09-14

**Authors:** Da Y. Park, Aung H. Naing, Trinh N. Ai, Jeung-Sul Han, In-Kyu Kang, Chang K. Kim

**Affiliations:** ^1^Department of Horticultural Science, Kyungpook National University Daegu, South Korea; ^2^School of Agriculture and Aquaculture, Tra Vinh University Tra Vinh, Vietnam

**Keywords:** antioxidant activity, relative fresh weight, relative gene expression, nano-silver, sucrose

## Abstract

We investigated the effects of sucrose and nano-silver (NAg) on extending the vase life of cut carnation flowers “Edun”. Sucrose (pulse treatment) suppressed ethylene production by downregulating the genes that code for its biosynthesis. Relative to the control, however, sucrose significantly promoted xylem blockage on cut stem surfaces and reduced relative fresh weight, antioxidant activity, and cysteine proteinase inhibitor gene (*DcCPi*) expression. Consequently, the sucrose-treated flowers had shorter vase lives than the control. In contrast, NAg suppressed ethylene production in the petal, prevented xylem blockage in the cut stem surface, and improved all the aforementioned parameters. Therefore, NAg increased flower longevity. The most effective treatment in terms of longevity extension and parameter improvement, however, was the combination of NAg and sucrose. These results suggest that sucrose can suppress ethylene production but does not necessarily extend the vase life of the flower cultivar. The role of NAg in increasing cut carnation longevity is mainly to inhibit xylem blockage rather than suppress ethylene production, and the combined effect of NAg and sucrose is most effective at prolonging cut carnation vase life, likely due to their synergetic effects on multiple modes of action.

## Introduction

Carnation senescence is regulated by endogenous ethylene produced from the transcription of the ethylene biosynthesis genes coding for 1-aminocyclopropane-1-carboxylic acid (ACC) synthase (ACS) and ACC oxidase (ACO) (Park et al., 1992; van Doorn, 2004; Hoeberichts et al., 2007; Yuan et al., 2012). Sucrose delays floral senescence by downregulating ethylene biosynthesis genes. This mode of action has been largely exploited in carnation and many other cut flowers (Nichols, 1973; Mayak and Dilley, 1976; van Doorn, 2004). In cut carnation flowers, sucrose delayed the ethylene climacteric (Dilley and Carpenter, 1975; Sacalis and Lee, 1987) by suppressing the *ACO* and *ACS* genes in flower tissues (van Doorn, 2004; Verlinden and Garcia, 2004). Sucrose also delayed visible senescence in sweet pea by suppressing climacteric ethylene production (Ichimura and Suto, 1999). Another advantage of using sucrose is that it can serve as a substrate for respiration and cell wall synthesis and improve cut flower vase life (Halevy and Mayak, 1979), in addition, it can also prevent water loss from the petal during cut flower senescence by increasing the amount of osmotic solutes in the cells (van Doorn and Woltering, 2008).

The sucrose concentrations required to prepare flower vase solutions with the correct sugar–water balance must be optimized for each cultivar (Lu et al., 2010). In the vase solution, sucrose may promote the growth of microbes, which block xylem vessels, impede water uptake by flower stems, and cause premature petal wilting or senescence (van Doorn, 1997; Ranwala and Miller, 2009). The role of sucrose in prolonging cut flower vase life is not fully elucidated to date.

Recently, it has been demonstrated that nano-silver (NAg) increases the vase life of various cut flowers by inhibiting ethylene production and bacterial growth (Hassan et al., 2014; Li et al., 2017). Sucrose has also been combined with NAg or silver thiosulfate to increase the vase life of cut flowers (Mor et al., 1984; Mortazavi et al., 2011; Moradi et al., 2012). It has been demonstrated in many cut flowers that sucrose alone or in combination with NAg delays climacteric ethylene production. Nevertheless, the effects of these substances on antioxidant activity, xylem bacterial growth, and ethylene biosynthesis/petal senescence gene expression have not yet been investigated in detail. In our preliminary experiment, we observed that the carnation cultivar “Edun” (*Dianthus caryophyllus* L.) produced moderate level of ethylene concentration, but its vase life lasted approximately 14 days (unpublished data). Hence, it is of interest to investigate the mechanism by which factors mainly affected vase life of this cultivar.

In this study, we investigated the individual and combined effects of sucrose and NAg on cut flower senescence. We measured the relative fresh weight (RFW) of cut carnation flowers and determined other parameters associated with flower senescence, such as xylem vessel blockage, ethylene production, ethylene biosynthesis-related gene expression, and antioxidant activity.

## Materials and Methods

### Plant Material

The flower stems of carnation cultivar “Edun” (*D. caryophyllus* L.), a large flowered cultivar (with pinky white color) that grows with one big flower on every stem, used in this experiment were freshly cut in a greenhouse during March and April of 2016. The greenhouse is set with the favorable environmental factors, such as temperature (at 22–24°C during the day and 16°C during night), light (14 h photoperiod with the help of supplemental lighting, approximately 45.36 molm^-2^ d^-1^), and relative humidity (RH, 70%), for production of quality cut flowers. Therefore, preharvest conditions that negatively affect the keeping quality of cut flowers (e.g., 24 h photoperiod and RH above 85%) were avoided (Fanourakis et al., 2013, 2015; van Meeteren and Aliniaeifard, 2016), Upon arrival the laboratory, the cut flower stems were placed in distilled water and re-cut to achieve a length of about 40 cm, in accordance with the commercial practice. In addition, leaves that were in direct contact with the vase water were carefully removed by hand.

### Treatment with Sucrose

In this study, we used the same sucrose concentrations to validate their positive effects on cut flower vase life. The initial fresh weights of all cut flowers were recorded. Those of approximately equal fresh weight were placed in 1.5-L plastic bottles containing sucrose (0, 58, and 146 mM) dissolved in 500 mL distilled water for 24 h. Next, the treated stems were thoroughly rinsed under tap water and placed in vases containing 500 mL fresh distilled water (without sucrose). The vases were maintained in a growth chamber at 20 μmol^-2^ s^-1^ (fluorescent tubes) light intensity, 23°C, and 60–70% RH for 12 h. Each replication contained 15 cut flower stems. There were three replicates, and the experiment was conducted in triplicate.

On day 3, five flowers from each replication were selected to determine the RFW and vase life. The remaining 10 flowers in each replication were used to isolate bacteria, investigate xylem vessel blockage, estimate ethylene production, analyze ethylene biosynthesis and senescence gene transcription, and measure antioxidant activities.

### Effect of NAg Alone or in Combination with Sucrose on the Vase Life of the Carnation

We investigated the effects of NAg alone or in combination with sucrose on cut flower vase life. Flower stems of similar fresh weight were placed in vases containing 500 mL distilled water (control), NAg with the size of 60 nm (Sigma–Aldrich) (0.23 mM) alone, or a combination of NAg and 58 mM sucrose. The vases were maintained in the same growth chambers and conditions as the previous experiment. Physiological and molecular analyses were also performed as before. Once again, each replication contained 15 cut flower stems and there were three replicates. Each experiment was conducted in triplicate.

### Vase Life and RFW

The postharvest life of each flower was assessed when more than one-third of its petals showed inward rolling, browning, or loss of ornamental value (petal senescence and discoloration). The fresh weight of each cut stem was measured at 3–4 days intervals. The RFW was calculated as follows: RFW (%) = (FW_t_/FW_0_) × 100; where FW_t_ is the fresh weight of the stem (g) on days 3, 6, 10, or 14, and FW_0_ is the initial fresh weight of the stem (g) on day 1 (He et al., 2006). Five flowers were used per treatment, with three replicates.

### Measurement of Ethylene

Five grams of petals from each treatment were weighed and sampled on days 3, 6, 10, and 14. They were placed in a 50-mL glass tube which was then sealed with a rubber septum. The petals were maintained for 16 h at 20°C. A 1-mL aliquot of the gas in the headspace was withdrawn through the septum using a 1-mL syringe. The gas was analyzed with a gas chromatograph (GC-2010, Shimadzu) calibrated to detect ethylene. Three syringes (replicates) were used for each treatment.

### RNA Extraction and qRT-PCR Analysis

Total RNA was extracted from 100 mg of petals collected on days 3, 6, 10, and 14 using the RNeasy Plant Mini Kit (Qiagen, Hilden, Germany). The cDNA was synthesized from 1 μg total RNA using an oligo dT_20_ primer and a reverse transcription kit (ReverTra Ace-á, Toyobo, Japan). The transcript levels of 1-aminocyclopropane-1-carboxylate oxidase (*DcACO1*), 1-aminocyclopropane-1-carboxylate synthase (*DcACS1*), and the petal senescence-resistance genes (cysteine proteinase inhibitor gene *DcCPi*) were measured using a StepOnePlus Real-Time PCR system (Thermo Fisher Scientific, Waltham, MA, United States). To confirm the amount of template RNA, fragments of the carnation reference genes (*DcUbq*4-5 and *DcACT*) were used as internal controls. The primers and PCR conditions used for detecting the expression levels of these genes are listed in **Table 1**. The method used to calculate relative gene expression was quantitation–comparative method *C*_T_ (ΔΔ*C*_T_). Five samples per treatment were used, and each analysis was repeated three times.

**Table 1:** Primer sequences used in qRT-PCR for detecting genes related to ethylene production and petal senescence.

Gene	Primer sequence (5′–3′)	PCR condition
*ACO1*	F: CCG AGC AAC TGT TGG ACT TG	
	R: AGA GAA TGA TGC CAC CAG CG	
*ACS1*	F: TCC AGG GTT TAG GGT TGG GA	95°C (10 min) → [95°C (15 s) → 57°C (1 min) → 72°C (35 s)] × 40 cycles → 95°C (15 s) → 59.3°C (1 min) → 95°C (15 s)4
	R: CCT TCC TAC AAA CGC CTC GT	
*CPi*	F: GGT GAA ACC GTG GGT GAA CT	
	R: CCT TCC AGA AAC ATG CTC CG	
*Actin*	F: GCA CGG TAT CGT CAC CAA CT	
	R: AGC CTT TGG GTT AAG AGG CG	
*Ubq4*	F: TTGGTTTCAGGGCTGGTTTG	
	R: CACAGAGTACTGACACGATAA ACATCAA	
*Ubq5*	F: CTCCACCTTGTCCTCCGTCTT	
	R: CCAGCCCTGAAACCAACAAC	

### Determination of Antioxidant Activities

Petals from the sucrose and NAg treatments were collected on days 15 and 17, respectively, when the flowers had lost their ornamental value. These petals were frozen for analysis of antioxidant activities. To determine the activities of 1,1-diphenyl-2-picrylhydrazyl radical (DPPH) and 2,2′-azino-bis-3-ethylbenzthiazoline-6-sulphonic acid (ABTS), 5 g frozen petals were used and analyzed according to the method of Kim et al. (2014). Five samples were used per treatment, and each analysis was repeated three times.

### Xylem Blockage Examination by SEM

Xylem blockage was investigated using scanning electron microscopy (SEM; JEOL Ltd., Tokyo, Japan). Stem segments 3 mm long were excised with scalpel blades from the base of each stem on days 0 (starting day after treatment), 6, and 15 for the sucrose treatment and the NAg treatment. The stem segments were immediately fixed in formalin–acetic acid–alcohol and kept overnight according to the protocol of Naing et al. (2015). The samples were then dehydrated for 10 min using serial ethanol concentrations (25, 50, 70, 85, and 100%). The dehydrated samples were then dried to the critical point at room temperature, coated with gold-palladium on a Quick Cool Coater (Sanyu-Denshi, Japan), examined under the SEM and photographed.

### Statistical Analysis

The data were analyzed using SPSS v. 11.09 (IBM Corporation, Armonk, NY, United States) and presented as means of three replicates. Duncan multiple range test (DMRT) and least significant difference (LSD) test were used to identify the differences between means. The significance level was set at *P* < 0.05.

## Results

### Effects of Different Concentrations of Sucrose on RFW and Vase Life

**FIGURE 1:**
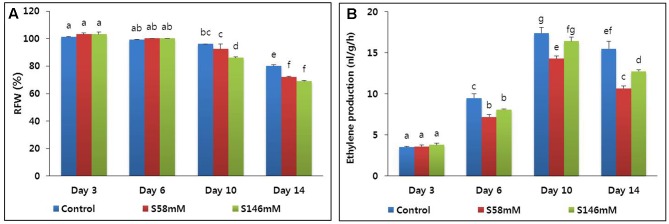
Effect of sucrose on relative fresh weight (RFW) **(A)** and ethylene production **(B)** of flowers of cut carnation “Edun” during the vase period. Data represent mean of three replicates, while bar indicates standard deviation. Means with different letters are significantly different (DMRT, *p* < 0.05).

The RFW of all flowers was the highest by day 3 and did not decline until day 6. By day 10, however, the RFW of all flowers started to decline. Thereafter, the RFW of the sucrose-treated flowers decreased faster than those of the control (**Figure 1A**). Nevertheless, there was no significant difference between treatments in terms of the rates of decline in RFW by day 14. The rapid drop in RFW also caused premature petal senescence, which was first observed in the sucrose-treated flowers on day 13. By day 15, the flower vase life ended (**Table 2**). In contrast, the vase life of the control ended on day 17. Therefore, the sucrose treatment reduced carnation RFW and vase life.

**Table 2:** Effect of different concentrations of sucrose on vase life of the cut carnation flower “Edun”.

Treatment	Vase life (days)
Control	17a
Sucrose 58 mM	15b
Sucrose 146 mM	15b

*Data represent mean of three replicates. Means with different letters are significantly different (LSD test, *p* < 0.05)*.

### Ethylene Production and its Related Gene Expression

**FIGURE 2:**
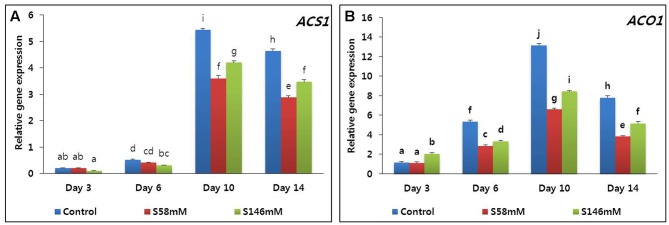
Effect of sucrose on transcript levels of 1-aminocyclopropane-1-carboxylate synthase (*DcACS1*) **(A)** and 1-aminocyclopropane-1-carboxylate oxidase (*DcACO1*) **(B)** genes in the petals of “Edun” carnation. Data represent mean of three replicates, while bar indicates standard deviation. Means with different letters are significantly different (DMRT test, *p* < 0.05).

By day 3, very little ethylene was released from the flowers. The quantities produced among the treatments did not significantly differ. By day 6, ethylene production increased, especially in the control, and reached a peak on day 10 (**Figure 1B**). In contrast, the sucrose treatments suppressed ethylene production to a level below that of the control. By day 14, ethylene production had decreased in all treatments. The 58 mM more effectively suppressed ethylene production than did the 146 mM sucrose treatment. The quantitative real-time polymerase chain reaction (qRT-PCR) analysis indicated that the transcript levels of the ethylene biosynthesis genes (*ACS1* and *ACO1*) were lowest on day 3. They increased slightly on day 6, reached maxima on day 10, and decreased on day 14 (**Figures 2A,B**). After day 6, the transcript levels were lowest with the 58 mM treatment, followed by the 146 mM then the control.

### Extent of Petal Senescence and its Related Gene Expression

**FIGURE 3:**
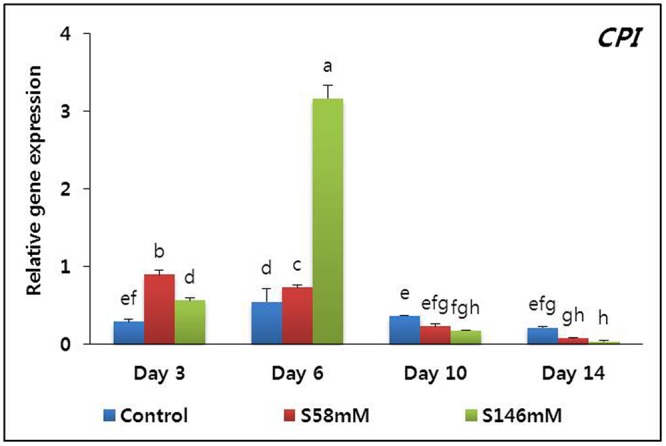
Effect of sucrose on the transcript levels of cysteine proteinase inhibitor (*DcCPi*) gene in the petals of “Edun” carnation. Data represent mean of three replicates, while bar indicates standard deviation. Means with different letters are significantly different (DMRT test, *p* < 0.05).

On day 3, the flowers treated with sucrose appeared fresher than those of the control. The sucrose-treated flowers attained a peak of freshness by day 6. Thereafter, their appearance resembled that of the control flowers. By day 10, however, the RFW of the sucrose-treated flowers was slightly lower than that of the control. Petal senescence was apparent by day 15, particular for the 146 mM sucrose treatment. Senescence was less obvious in the petals treated with 58 mM sucrose and absent in the control flowers (data not shown). The senescence-resistance gene (*DcCPi*) transcript levels were higher in the sucrose-treated flowers than the control on days 3 and 6 but fell to a level below that of the control by day 10. The lowest *DcCPi* transcript levels were observed in the sucrose-treated flowers on day 14 (**Figure 3**).

### Antioxidant Activities

**FIGURE 4:**
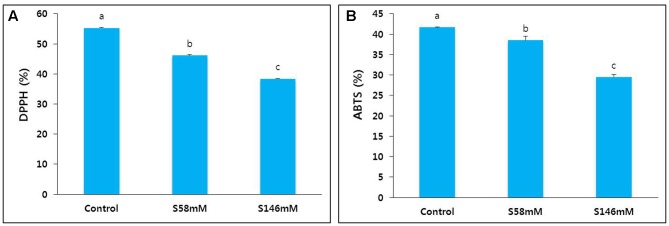
Effect of sucrose on DPPH activity **(A)** and ABTS activity **(B)** in the petals of “Edun” carnation. Data were collected on day 15 after treatment. Data represent mean of three replicates, while bar indicates standard deviation. Means with different letters are significantly different (LSD test, *p* < 0.05).

In this study, we collected petals from sucrose-treated flowers on day 15 when they showed senescence. We determined their DPPH and ABTS activities and compared them with those of the control. These activity levels (**Figures 4A,B**) reflected the extent of petal senescence because they were higher in the control than the sucrose treatments, and higher in the 58 mM sucrose treatment than the 146 mM sucrose treatment.

### Scanning Electron Microscopy

**FIGURE 5:**
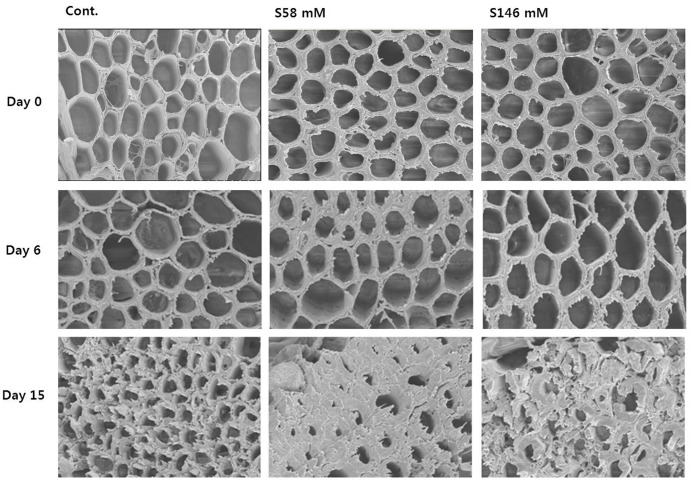
Comparison of cut flower stem surfaces in control, 58 mM sucrose, and 146 mM sucrose solutions on days 0, 6, and 15 after treatment.

Throughout the experiment, we examined the flower stem xylem, which is mainly responsible for water transport to the flowers. On day 6, when the RFW was still relatively high, little, or no xylem blockage was detected in any of the treatments (**Figure 5**, middle row) as observed in day 0 (**Figure 5**, upper row). By day 10, apparent xylem blockages were observed in all cut stem surfaces, but were greater in the sucrose treatments than the control (data not shown). By day 15, the xylem blockages were substantial enough to prevent water transport to the flowers and cause an earlier onset of petal senescence than the control (**Figure 5**, lower row).

### Effect of NAg on RFW and Vase Life

**FIGURE 6:**
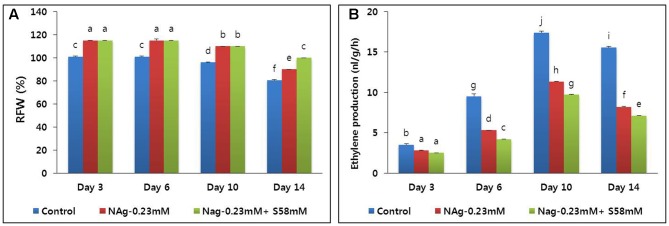
Effect of NAg alone and in combination with sucrose on relative fresh weight (RFW) **(A)** and ethylene production **(B)** of flowers of cut carnation “Edun” during the vase period. Data represent mean of three replicates, while bar indicates standard deviation. Means with different letters are significantly different (DMRT, *p* < 0.05).

When the cut flower stems were treated with 0.23 mM NAg, their RFW were significantly higher than those of the control for the duration of the vase life. Both the control and the NAg-treated flowers maintained constant RFW until day 6, but they started to decline steadily thereafter (**Figure 6A**). On day 14, the RFW were significantly reduced in the control flowers. In the 0.23 mM NAg plus 58 mM sucrose treatment, the RFW did not significantly differ from those of the 0.23 mM NAg treatment until day 10, at which time the RFW fell slightly for both treatments. By day 14, however, the RFW of the combined 0.23 mM NAg plus sucrose 58 mM treatment were significantly higher than those of the 0.23 mM NAg treatment. Both the RFW and the flower vase life were highest in the combined treatment, followed by the 0.23 mM NAg treatment, then the control (**Table 3**).

**Table 3:** Effect of nano-sliver (NAg) alone or in combination with sucrose on vase life of the cut carnation flower “Edun”.

Treatment	Vase life (days)
Control	17c
NAg (0.23 mM)	21b
NAg (0.23 mM) + sucrose 58 mM	23a

*Data represent mean of three replicates. Means with different letters are significantly different (LSD test, *p* < 0.05)*.

### Ethylene Production and its Related Gene Expression

**FIGURE 7:**
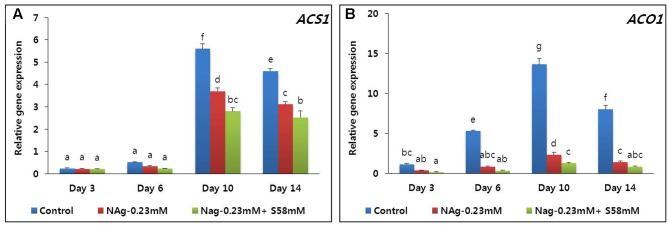
Effect of NAg alone or in combination with sucrose on transcript levels of 1-aminocyclopropane-1-carboxylate synthase (*DcACS1*) **(A)** and 1-aminocyclopropane-1-carboxylate oxidase (*DcACO1*) **(B)** genes in the petals of “Edun” carnation. Data represent mean of three replicates, while bar indicates standard deviation. Means with different letters are significantly different (DMRT, *p* < 0.05).

On day 3, all ethylene production rates were relatively low, but that of the control was significantly higher than those of the sucrose treatments. The ethylene production increased in all treatments by day 10 but was highest in the control (**Figure 6B**). By day 14, all ethylene production levels declined, but that of the control was significantly higher than those of the two treatments. Throughout the vase period, the combined treatment more effectively suppressed ethylene production than the single treatment. Transcriptional analysis of the ethylene biosynthesis genes (*ACS1* and *ACO1*) via qRT-PCR indicated that the transcript levels in the combined treatment were the lowest, followed by the NAg treatment, then the control (**Figures 7A,B**).

### Petal Senescence and its Related Gene Expression

**FIGURE 8:**
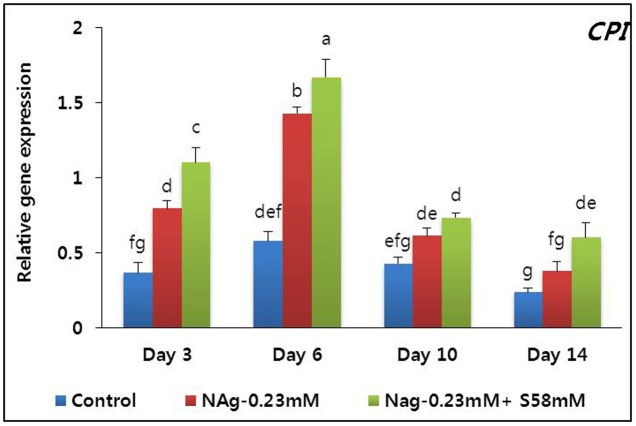
Effect of NAg alone or in combination with sucrose on the transcript levels of cysteine proteinase inhibitor (*DcCPi*) gene in the petals of “Edun” carnation. Data represent mean of three replicates, while bar indicates standard deviation. Means with different letters are significantly different (DMRT, *p* < 0.05).

Petal senescence was not observed in any treatment until day 14. Flower buds opened sooner in the NAg and combination treatments than the control. The flowers in the NAg and combination treatments were larger and remained fresh longer than those of the control. The petal anthocyanin content reached a peak on day 6 and was highest with the combination treatment followed by the NAg treatment, then the control (data not shown). Although apparent petal senescence was not observed until day 14, the RFW significantly decreased in all treatments, especially in the control. Analysis of the senescence-resistance gene (*DcCPi*) indicated that in all treatments, its expression reached a maximum on day 6, and it continued to be expressed until day 14, when its expression level rapidly declined (**Figure 8**). As predicted, its expression level was highest in the combination treatment, followed by the NAg treatment, and then the control.

### Antioxidant Activities

**FIGURE 9:**
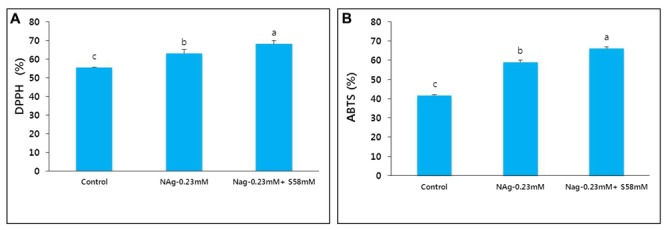
Effect of NAg alone or in combination with sucrose on DPPH activity **(A)** and ABTS activity **(B)** in the petals of “Edun” carnation. Data were collected on day 17 after treatment. Data represent mean of three replicates, while bar indicates standard deviation. Means with different letters are significantly different (LSD test, *p* < 0.05).

The activities of the antioxidants DPPH and ABTS were positively correlated with flower vase life. By day 17, the control flower vase life ended and the DPPH and ABTS activities were significantly reduced. In contrast, the NAg and combination treatments extended flower vase life to day 21 and day 23, respectively, because the antioxidant activities in the treated flowers were significantly higher than those in the control flowers (**Figures 9A,B**). The antioxidant activity levels were negatively correlated with the extent of petal senescence.

### Scanning Electron Microscopy

**FIGURE 10:**
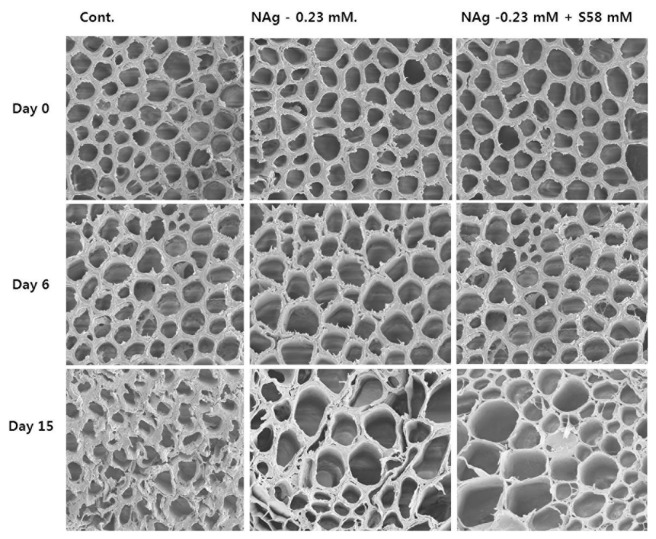
Comparison of cut flower stem surfaces in control, 0.23 mM NAg, and 0.23 mM plus 58 mM sucrose solutions on day 17 after treatment.

We used SEM to investigate the association between xylem status and vase life in each treatment. The xylem statuses observed in all treatments were similar on days 0 and 6 (**Figure 10**, upper and middle rows). However, as predicted, the control flowers, whose vase life was shorter than those of the other treatments, had greater xylem blockage on their stem surfaces than those of the treated flowers on day 15 (**Figure 10**, lower row). In addition, by day 21, when the vase life of the NAg-treated flowers ended, the stem xylems were more blocked than those of the flowers receiving the combination treatment.

## Discussion

The efficacy of sucrose in delaying cut flower petal senescence has been previously reported for lily, tree peony, sweet pea, carnation, and orchid (Ichimura and Suto, 1999; Chen et al., 2001; Verlinden and Garcia, 2004; Hoeberichts et al., 2007; Arrom and Munne-Bosch, 2012; Zhang et al., 2012). These studies indicated that sucrose suppressed ethylene production and delayed petal senescence by downregulating the genes involved in ethylene biosynthesis and petal senescence; in addition, they claimed that sucrose maintained flower fresh weight as well by closing petal stomata and reducing water loss. As expected, in the present study, the sucrose concentrations (58 or 146 mM) suppressed ethylene production in the carnation by downregulating ethylene biosynthesis genes and also maintained the flower RFW until day 6 but did not extend the vase life of the cultivar tested. However, the vase life of the sucrose-treated flowers was shorter than that of the control, which was not consistent with the studies of Pun et al. (2016) and Verlinden and Garcia (2004) because they claimed that these concentrations extended vase life of carnation. In fact, sucrose serves as a substrate for respiration and cell wall synthesis and it maintains water balance, all of which should prolong vase life. In the present study, however, the flower RFW declined in the sucrose treatment after day 10 and the rate of decrease thereafter was significantly higher than that observed in the control. Moreover, the water uptake rate and volume were lower and the vase solution was a deeper yellow color in the sucrose treatment than the control, which would be due to presence of bacteria in the vase solution (van Doorn, 1997; Ranwala and Miller, 2009). In addition, the senescence-resistance gene (*DcCPi*) transcription level and the activities of the antioxidants DPPH and ABTS, all of which are positively correlated with cut flower vase life (Droillard and Paulin, 1987; Kumar et al., 2008; Zeng et al., 2011; Tanase et al., 2013, 2015; Dwivedi et al., 2016) were lower in the sucrose treatments than the control.

In this study, although sucrose suppressed ethylene production in the carnation flowers by downregulating gene expression relative to the control, on the other hand, it shortened flower vase life by decreasing the RFW, increasing petal senescence, and reducing senescence-resistance gene expression, antioxidant activity, and water uptake. We therefore examined the status of the cut stem surfaces and xylem vessels by SEM on days 0, 6, and 15 to understand why water uptake was lower and petal senescence faster in the sucrose treatment than the control. By days 0 and 6, xylem blockages on the cut stems in all treatments were not obvious. For this reason, the flower RFW and the cut stem water uptake were the highest for all treatments on days 3 and 6; in addition, the RFW was not significantly different among the treatments. Nevertheless, by day 15, the cut stem surfaces and xylem vessels were blocked, and more severely in the sucrose treatment than the control. The reason is that, over time, the sucrose enhanced bacterial growth in the vase solution and on the cut stem surfaces (van Doorn, 1997; Ranwala and Miller, 2009). This blockage prevented water transport to flowers, reduced the RFW, and induced early petal senescence. The association between xylem blockage and vase life has been reported for rose, gerbera, and gladiolus (Liu et al., 2009; Li et al., 2012, 2017). Lethal wilting related to hampered water uptake, owing to xylem vessel blockage of the stem cut end, is aggravated in cut flowers with uncontrolled rates of water loss (Fanourakis et al., 2012). Decreased transpirational water loss by means of either very responsive stomata (Fanourakis et al., 2012) or employment of antitranspirant compounds (Fanourakis et al., 2016) has been shown to partly alleviate lethal wilting symptoms by maintaining a positive water balance for longer periods.

Sucrose may suppress ethylene production by downregulating ethylene biosynthesis genes. Nevertheless, the effectiveness of sucrose at prolonging flower vase life may be explained primarily by its ability to control water uptake. The ineffectiveness of sucrose in prolonging the vase life of flowers that are resistant to ethylene has been reported elsewhere (van Doorn and Stead, 1994; van Doorn, 2004). Shahri et al. (2010) reported that the efficacy of sucrose at extending the vase life of different cultivars in the same family varied with the ethylene sensitivity of each cultivar. The vase lives did not significantly differ between the two sucrose treatments. Nevertheless, ethylene suppression and antioxidant activities were lower in the 146 mM treatment than the 58 mM treatment. At the higher sucrose concentration, the elevated sugar content in the cut flower stems would promote excessive bacterial growth there, and in the vase solution as well. Zhang et al. (2012) reported that high sucrose concentrations (60 and 90 g L^-1^) prevented water uptake in tree peony flowers.

Recently, it has been shown that NAg is an antimicrobial agent that inhibits bacterial growth in cut flower stem xylem, thereby increasing water uptake and vase life (Liu et al., 2009; Li et al., 2012, 2017). We tested the effect of NAg alone or in combination with sucrose on the vase life of the cultivar used in the present study. As expected, NAg treatments effectively suppressed xylem blockage on the cut stem surfaces until day 15 and extended vase life longer than that of the control. In addition, NAg increased the RFW, antioxidant activities, and *DcCPi* gene expression, and suppressed ethylene production by downregulating ethylene biosynthesis genes. The suppression of ethylene production by NAg is consistent with the results reported for lily and rose by Kim et al. (2005) and Hassan et al. (2014). In addition, one possible explanation for the ethylene suppression by NAg would be that sliver blocks the activity of ethylene by reducing receptor capacity to bind ethylene (Beyer, 1976), and ACC, an ethylene precursor that promotes ethylene production (Bleecker and Kende, 2000). The combination treatment suppressed ethylene production more than NAg alone because sucrose suppresses ethylene production in cut flowers. In addition, the RFW for the combination treatment was higher than that for NAg on day 14 since the latter treatment lacked sucrose, which is required for respiration, cell wall synthesis, and osmotic balance in flowers (Halevy and Mayak, 1979; van Doorn and Woltering, 2008). The combination treatment maintained the RFW, petal freshness, and antioxidant activity longer than did NAg alone. Although sucrose did not extend cut flower vase life, it significantly extended vase life longer in combination with NAg than did NAg alone. Therefore, in terms of lengthening the vase life of this carnation cultivar, there is a synergy between these two substances.

## Conclusion

Sucrose treatments significantly suppressed ethylene production in cut carnation flowers by downregulating ethylene biosynthesis genes. Nevertheless, they reduced cut flower longevity compared to that of the control by causing xylem blockage on the cut stem surfaces, and by lowering senescence-resistance gene expression and antioxidant activity. In contrast, NAg significantly increased cut flower longevity compared to the control by effectively suppressing xylem blockage on the cut stem surface, and by increasing all of the parameters analyzed. All of the factors associated with cut flower longevity improved (relative to the control) when sucrose was combined with NAg. This study demonstrates that sucrose and NAg function synergistically in extending cut carnation flower longevity and would, therefore, be helpful in maintaining a high cut flower quality.

## Author Contributions

AN and DP designed the study, conducted the experiments, and wrote the manuscript. CK supervised experiments at all stages and performed critical revisions of the manuscript. I-KK, TA, and J-SH assisted with experimental procedures and data analysis. All authors read and approved the final manuscript.

## Conflict of Interest Statement

The authors declare that the research was conducted in the absence of any commercial or financial relationships that could be construed as a potential conflict of interest.
